# Truth is in the eye of the beholder: Perception of the Müller-Lyer illusion in dogs

**DOI:** 10.3758/s13420-018-0344-z

**Published:** 2018-09-05

**Authors:** Benjamin Keep, Helen E. Zulch, Anna Wilkinson

**Affiliations:** 1 School of Life Sciences, Joseph Banks Laboratories, Green Lane, University of Lincoln, Lincoln, LN6 7DL UK; 2 Dogs Trust, London, UK

**Keywords:** Dog, Canine, Cognition, Perception, Visual discrimination, Visual illusion

## Abstract

Visual illusions are objects that are made up of elements that are arranged in such a way as to result in erroneous perception of the objects’ physical properties. Visual illusions are used to study visual perception in humans and nonhuman animals, since they provide insight into the psychological and cognitive processes underlying the perceptual system. In a set of three experiments, we examined whether dogs were able to learn a relational discrimination and to perceive the Müller-Lyer illusion. In Experiment 1, dogs were trained to discriminate line lengths using a two-alternative forced choice procedure on a touchscreen. Upon learning the discrimination, dogs’ generalization to novel exemplars and the threshold of their abilities were tested. In the second experiment, dogs were presented with the Müller-Lyer illusion as test trials, alongside additional test trials that controlled for overall stimulus size. Dogs appeared to perceive the illusion; however, control trials revealed that they were using global size to solve the task. Experiment 3 presented modified stimuli that have been known to enhance perception of the illusion in other species. However, the dogs’ performance remained the same. These findings reveal evidence of relational learning in dogs. However, their failure to perceive the illusion emphasizes the importance of using a full array of control trials when examining these paradigms, and it suggests that visual acuity may play a crucial role in this perceptual phenomenon.

The class of visual objects known as visual illusions are characterized by the fact that the elements of which they are composed have an arrangement that leads to an erroneous perception of their physical aspects (Coello, Danckert, Blangero, & Rossetti, 2007). Visual illusions are used to study visual perception in humans and occur automatically, meaning that subjects cannot stop themselves from perceiving the illusion even with prior knowledge that it is an illusion (Feng, Chouinard, Howell, & Bennett, 2017). These illusions provide insight into the psychological and cognitive processes underlying visual perception (Kelley & Kelley, 2014).

Perception of visual illusions could be advantageous for nonhuman animals (hereafter simply *animals*) during courtship, mate choice, and competition (Sovrano, Pos, & Albertazzi, 2015) when the relative size of an object is important—for example, in the judgment of male claw length by female fiddler crabs (*Uca mjoebergi*) during mate choice (Callander, Jennions, & Backwell, 2011). Visual illusions provide a useful means for investigating species similarities and differences in visual processing (for a review, see Feng et al., 2017; Rosa Salva, Sovrano, & Vallortigara, 2014). In recent years, research into visual illusions in animals has received much attention; such studies have included those into illusory motion in cats, fish, and rhesus monkeys (Agrillo, Gori, & Beran, 2015; Bååth, Seno, & Kitaoka, 2014; Gori, Agrillo, Dadda, & Bisazza, 2014); the Zöllner illusion in pigeons and bantams (Watanabe, Nakamura, & Fujita, 2011, 2013); the Ponzo illusion in pigeons and primates (Fujita, 1997; Fujita, Blough, & Blough, 1993); the corridor illusion in baboons (Barbet & Fagot, 2002); and the Ebbinghaus–Titchener illusion in baboons, pigeons, a dolphin, and domestic chicks (Murayama, Usui, Takeda, Kato, & Maejima, 2012; Nakamura, Watanabe, & Fujita, 2008; Parron & Fagot, 2007; Rosa Salva, Rugani, Cavazzana, Regolin, & Vallortigara, 2013). The results obtained from these studies have been interesting, with some animals’ responses paralleling those of humans, whereas others have not.

The Ebbinghaus–Titchener illusion consists of two circles of equal size, one of which is surrounded by smaller circles, which appears larger to humans, and one of which is surrounded by larger circles, which appears smaller. During investigation of this illusion, a dolphin was found to be susceptible (Murayama et al., 2012)—that is, it responded to the illusion in the same way that humans do—whereas baboons showed no susceptibility (Parron & Fagot, 2007). In addition, pigeons were found to be susceptible to the illusion, but in a reversed direction relative to humans (Nakamura et al., 2008). This suggests differences in the perceptual processing of visual stimuli across species.

To our knowledge, three visual illusions have been investigated in the domestic dog: the Ponzo illusion, the Ebbinghaus–Titchener illusion, and the Delboeuf illusion (Byosiere, Feng, Rutter, et al., 2017; Byosiere, Feng, Woodhead, et al., 2017; Miletto Petrazzini, Bisazza, & Agrillo, 2017). The Ponzo illusion was investigated using a two-choice discrimination paradigm over a series of four experiments (Byosiere, Feng, Rutter, et al., 2017). Although there were inconsistencies, at both the group and individual levels across all four experiments, taken as a whole the experiments showed little evidence to suggest susceptibility to the illusion. For the other two illusions, a spontaneous-preference paradigm (Miletto Petrazzini et al., 2017) and a trained two-choice discrimination procedure (Byosiere, Feng, Woodhead, et al., 2017) were used. Susceptibility to the Delboeuf illusion was not reported, whereas dogs were found to be susceptible to the Ebbinghaus–Titchener illusion (in the opposite direction from the one usually observed in humans and some other animals) and to illusory contours (Byosiere, Feng, Woodhead, et al., 2017). Primates have been found to be susceptible to both the Ponzo and Delboeuf illusions (Ponzo, in chimpanzees and Rhesus monkeys: Fujita, 1997; Delboeuf, in chimpanzees: Parrish & Beran, 2014; in rhesus and capuchin monkeys: Parrish, Brosnan, & Beran, 2015), suggesting a possible discontinuity between dogs and primates in the perceptual biases affecting size judgments.

Geometric illusions, such as the Müller-Lyer illusion, allow us to investigate how the perceptual systems of different species integrate local stimulus features within global stimulus information (Sovrano et al., 2015). In this illusion, two lines of equal length are presented side by side. One of the lines has inward-pointing arrows (> <) located at either end, and is usually perceived as longer by humans; the other line has outward-pointing arrows (< >) located at either end, and is usually perceived as shorter.

The Müller-Lyer illusion has been investigated in a wide range of species, including mammals (capuchin monkeys: Suganuma, Pessoa, Monge-Fuentes, Castro, & Tavares, 2007; rhesus monkeys: Tudusciuc & Nieder, 2010), birds (pigeons: Nakamura, Fujita, Ushitani, & Miyata, 2006; Nakamura, Watanabe, & Fujita, 2009; a grey parrot: Pepperberg, Vicinay, & Cavanagh, 2008), fish (bamboo sharks: Fuss, Bleckmann, & Schluessel, 2014; teleost fish: Sovrano et al., 2015), and insects (ants: Sakiyama & Gunji, 2013), and in most but not all of these studies, susceptibility to the illusion has been reported. In these instances, animals appeared to perceive the illusion in the same direction as humans.

One challenge associated with investigating the Müller-Lyer illusion in animals is that it is impossible to instruct them to attend only to the lines and to ignore the arrowheads when making their judgments of length. To perceive the illusion, animals have to be able to process the figures at a global level while focusing on local stimulus features (Sovrano et al., 2015), and since dogs have been found to process stimuli with a globally oriented bias (Mongillo, Pitteri, Sambugaro, Carnier, & Marinelli, 2016; Pitteri, Mongillo, Carnier, Marinelli, & Huber, 2014), they have the potential to perceive the illusion.

When making Müller-Lyer judgments, if animals discriminate each figure as a whole, as opposed to judging the perceived difference between the line lengths, then their responses would parallel those observed in humans. To assess the perception of this illusion (and be certain that animals are not merely selecting the overall longer or shorter stimulus, rather than being deceived by the illusion), it is essential to add additional controls to the study that allow us to determine whether the subjects’ choice is based on perceived line length or whether they are using alternative stimulus features to make the discrimination.

Gaining a clearer understanding of how dogs perceive the world is of particular interest, because of the role that dogs play within human society. Working dogs aid humans in a variety of crucial tasks, from guiding to detection. To fulfill their roles, they are required to learn very specific skills and then perform under potentially stressful conditions. Once we understand better how dogs perceive objects visually, we can structure training in order to facilitate learning (working from the dog’s rather than from the human’s perspective, should these differ). This will not only make training more efficient and effective, but potentially reduce attentional stress on the dog, which in turn is likely to feed into a more resilient dog that is better able to perform to the optimum level under stress.

Much of the current work on dog perception has focused on interspecies visual communication (e.g., Tauzin, Csík, Kis, & Topál, 2015; Wallis et al., 2015), auditory perception (e.g., Pongrácz, Szabó, Kis, Péter, & Miklósi, 2014), and odor perception (e.g., Wright et al., 2017); however, much less is known about visual processing. Investigations of hierarchal stimulus processing have demonstrated a trend toward global precedence in dogs, although with much individual variation (Mongillo et al., 2016; Pitteri et al., 2014), and there is evidence that dogs are able to discriminate between complex artificial visual stimuli (e.g., Albuquerque et al., 2016; Huber, Racca, Scaf, Virányi, & Range, 2013; Müller, Schmitt, Barber, & Huber, 2015; Range, Aust, Steurer, & Huber, 2008). This suggests that dogs’ ability to process this information is highly developed; however, it is not immediately apparent which aspects of stimuli dogs have been responding to during such studies.

Dogs have been shown to successfully discriminate relative size, when making quantity-based food judgments (Baker, Morath, Rodzon, & Jordan, 2012; Miletto Petrazzini & Wynne, 2016, 2017; Ward & Smuts, 2007) and assessing opponent group size during intergroup conflicts (Bonanni, Natoli, Cafazzo, & Valsecchi, 2011). They have also demonstrated numerical competence in numerosity tasks (Macpherson & Roberts, 2013; West & Young, 2002). Whether they can discriminate on the basis of the continuous quantity of length is unknown. Thus, in Experiment 1 we investigated whether dogs were able to discriminate line length on the basis of a relational “longer than/shorter than” rule. Once the dogs had learned to discriminate vertical lines during Experiment 1, in the following two experiments we used modified versions of these lines (outward-pointing [< >] or inward-pointing [> <] arrowheads added to either end of each line) to assess perception of the Müller-Lyer illusion.

## Experiment 1

Dogs were trained to discriminate between lines of different lengths on the basis of a relative rule. They then completed a series of tests that investigated what they had learned and the thresholds of these abilities. Thus, they were presented with test stimuli in which lines differed in length but overall surface area was controlled. Following this, we tested their ability to generalize to novel pairings of trained stimuli, their ability to generalize the rule to entirely novel stimuli and, finally, we investigated the limits of their ability by decreasing the difference between the line lengths.

### Method

#### Subjects

Seven pet dogs (*Canis familiaris*) of various breeds took part in the experiment (Table 1). These were six females and one male that ranged in age from 1 to 9 years. Some of the dogs had previously experienced discrimination studies unrelated to the present experiment. The dogs were maintained on a normal feeding regime during testing (i.e., no changes to diet or feeding routine were made for the purposes of this study)

**Table 1:** Demographics of the experimental subjects

Name	Group	Breed	Age (years)	Sex	Neutered/Entire
Ivo	Long	Border Collie	9	M	E
Spook	Long	Border Collie	6	F	N
uMoya	Short	Labrador Retriever	1	F	E
Brimo	Short	Siberian Husky	5	F	N
Pan	Long	Siberian Husky	3	F	N
Mya	Short	Cocker Spaniel	6	F	N
Delta	Long	Belgian Shepherd	1	F	E

#### Apparatus

The apparatus consisted of a 15-in. monitor with an infrared touchscreen frame, encased in a wooden structure and attached to a computer. The software used was CognitionLab Light, version 1.9, build 57 (for further information on all aspects of this setup, please see Steurer, Aust, & Huber, 2012).

#### Stimuli

Twelve solid black vertical bars measuring 1.5–12.5 cm tall (1-cm increments) and 2 cm wide were used as the training stimuli. Pairs of these stimuli that differed in length by 4–11 cm (36 pairs) were used in the training trials. Each dog was pseudorandomly assigned 12 different pairs for the training phase. Stimuli were presented 15.5 cm apart. Despite the lines being different lengths, the active choice area (the area surrounding the stimulus upon which the animal could respond) was identical for each stimulus.

#### Procedure

Pretraining was based on the method used by Range et al. (2008). Dogs were shaped to nose-touch a stimulus presented in various positions on the touchscreen. After this, dogs had to successfully complete a series of discriminations using a two-alternative forced choice procedure before moving onto line length training.

##### Discrimination training

Dogs were trained using a two-alternative forced choice procedure. Each session started with the dog sitting ~ 2 m away from the touchscreen. The experimenter, who was familiar to the dogs, released the dog with a verbal command “go,” once the first set of stimuli were onscreen. Two line lengths were presented on the computer monitor, one of which was positive and the other negative. Choice of the positive stimulus resulted in a high-pitched tone and access to reinforcement. The reinforcement was placed by the experimenter in a bowl positioned behind the dogs starting position. Choice of the negative stimulus resulted in a low-pitched tone and a red screen (timeout) for 2–5 s; the exact time depended on a dog’s individual tolerance. This was followed by a correction trial (a repetition of the previous trial); correction trials continued until the animal had made the correct choice. All trials were separated by intertrial intervals of 2 s.

The stimuli were presented simultaneously, with the position of each stimulus pseudorandomized to ensure that animals received no more than three successive positive presentations on either side. Reward contingencies were counterbalanced across animals, with four of the dogs receiving reward for selecting the longer line, and three received reward for selecting the shorter line. To encourage relational learning, the stimulus pairings were arranged (where possible) so that a line could be positive in some pairings and negative in others. Each session consisted of 17 trials and animals received up to 11 sessions per day. Animals were considered to have learned the discrimination when they made 80% correct first choices in three consecutive sessions. If a dog stopped working at any point during a session, the session was stopped and the dog was given a break before resuming.

##### Control for surface area test

**Fig. 1:**
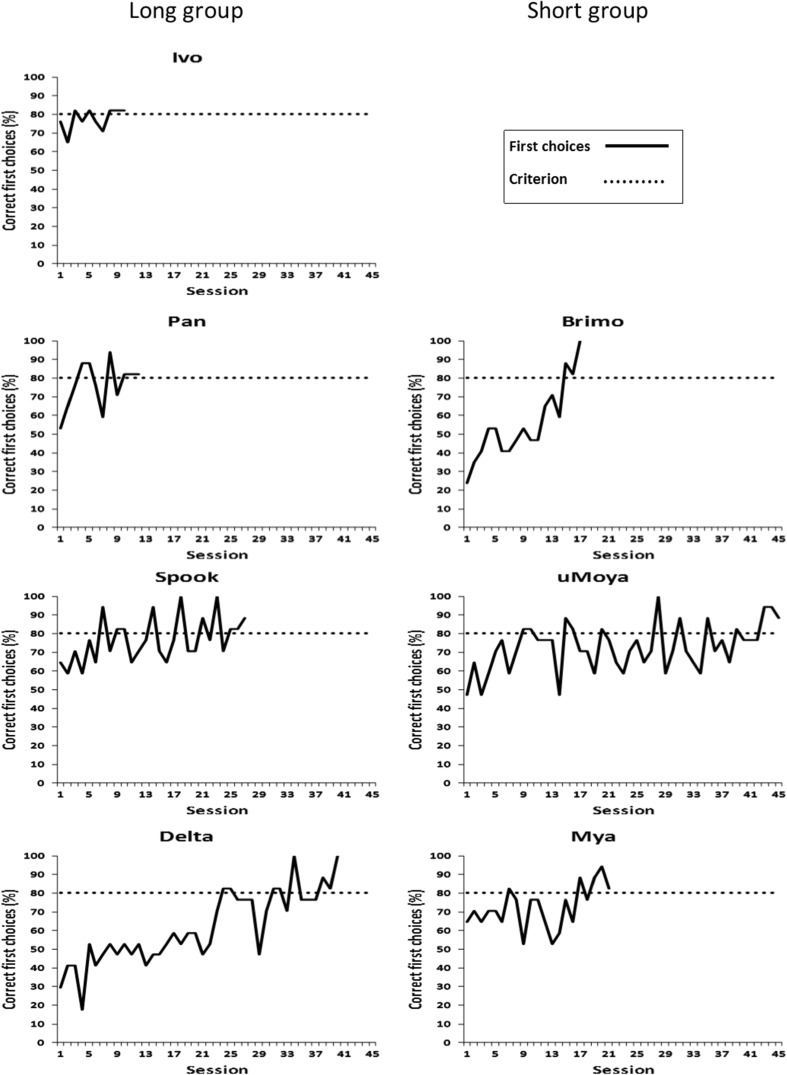
Experiment 1: Line length discrimination learning curves

To investigate whether the dogs were attending to the relative line length or overall surface area of the stimuli, they were tested using stimulus pairs that were matched for surface area but differed in length. During this test, each dog experienced all 12 lines used during training, which were 2 cm wide. Each line length was paired with a test stimulus that was either a longer, thinner line (1.5 cm wide) or a shorter, wider line (4 cm wide), resulting in pairs of stimuli that were equal in surface area but differed in length (Fig. 1). These stimuli were balanced across subjects, so that each line length was paired with equal numbers of thinner and wider lines. If the dogs were using surface area to make the discrimination, then we would expect them to perform at chance during these test trials.

Animals received 12 test stimuli presented over 18 sessions (four per session). Each test stimulus was presented six times, resulting in a total of 72 test trials. Test trials were pseudorandomly interspersed with the training trials, to ensure that test trials were never presented first, last, or consecutively. The test trials were identical to the training trials, except that animals received no differential reinforcement (they were never rewarded and did not receive a timeout during test trials, irrespective of choice). Only test data from sessions in which animals performed at greater than 80% correct first choices during the training trials were used for analysis. If performance was less than this, the session was repeated.

##### Generalization of relative line length rule test

To examine whether dogs were able to generalize their learning to novel stimulus pairings, they received test trials in which they were presented with four novel pairings (the stimuli within each pair, differed by 4–11 cm). Pair 1 consisted of two stimuli previously experienced individually but never in combination; Pair 2 contained one previously experienced stimulus and one novel stimulus; Pair 3 contained two novel stimuli; and Pair 4 contained a stimulus that had always been reinforced as longer or shorter, paired with a novel line length that reversed this contingency. Animals received six exposures to each pairing over the course of six sessions, resulting in a total of 24 test trials. The procedure was identical to that used in the control test for surface area.

##### Discrimination threshold test

To examine the limits of the dogs’ discrimination abilities, they were presented with nine randomly assigned test stimulus pairs with length differences (3.5, 3.0, 2.5, 2.0, 1.5, 1.0, 0.75, 0.5, and 0.25 cm), to test at which point they could no longer discriminate between two line lengths. Lines were presented in a random order within test sessions. Animals received six exposures to each pairing over 18 sessions, resulting in a total of 54 test trials. The procedure was identical to that used in the control test for surface area.

#### Data analysis

The data were checked for normality using Shapiro–Wilk tests. The discrimination training data were reported as the mean number of training sessions (± standard error) to meet criterion. The comparison of performance between the animals rewarded for the longer or the shorter line lengths during training was assessed using a Mann–Whitney *U* test. Discrimination performance during the tests is reported as the mean percent correct ± standard error, and the test trials were assessed using one-sample Poisson rate tests, which compared responses to chance level (.5). The data were analyzed using SPSS version 22 and Minitab 17.0.

### Results

#### Discrimination training

**Fig. 2:**
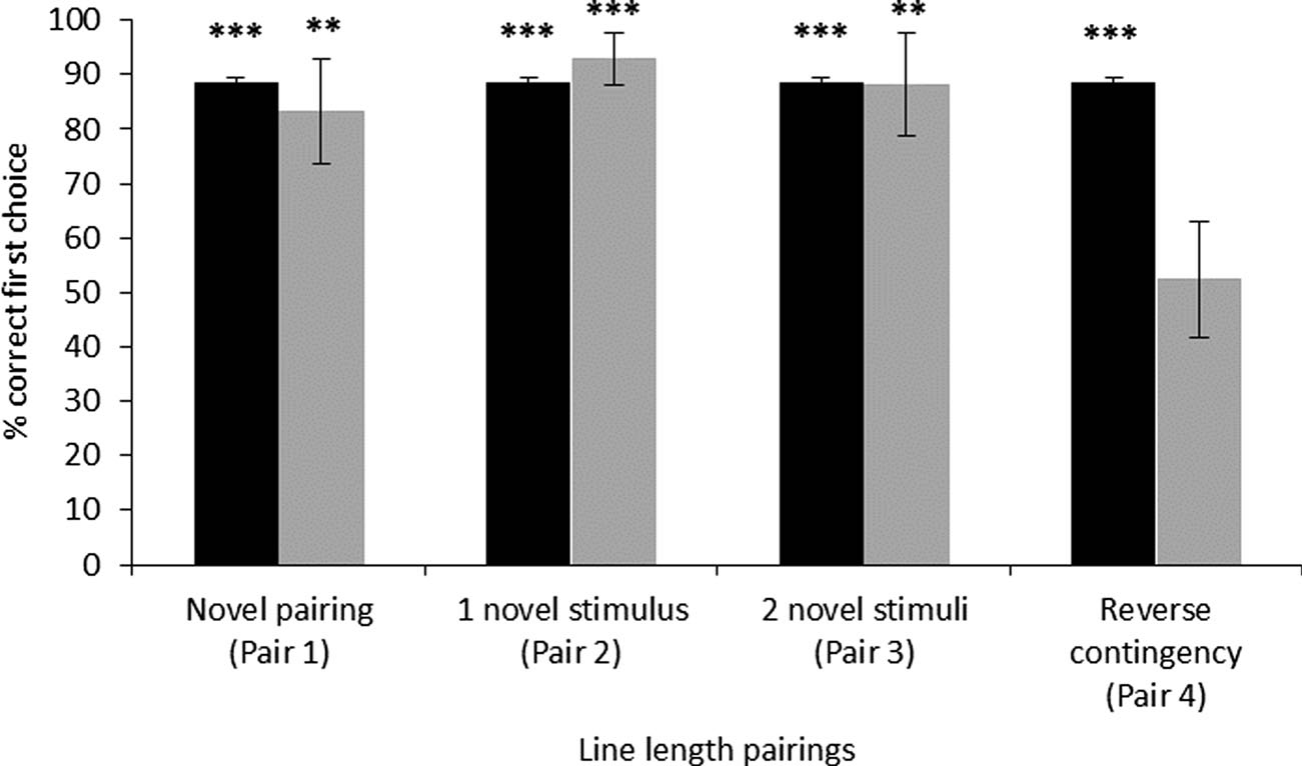
Mean ± *SE* (%) correct first choices for the generalization test. The black bars show dogs’ performance on the training trials during probe testing, and the gray bars depict dogs’ performance on the probe trials. One-sample Poisson rate test: ^**^p < .01, ^***^p < .001

All seven dogs reached the learning criterion, and they took an average of 24.6 ± 5.2 training sessions to do so. There was no difference in performance between dogs that were reinforced for choosing the longer or the shorter stimulus (Mann–Whitney *U* = 4, *p* = .629; Fig. 2).

#### Control test for surface area

The data from the control test for surface area revealed that dogs chose on the basis of relative line length rather than surface area, with an accuracy of 65.7% ± 4.7, which is significantly different from chance (*p* < .001). This suggests that the dogs learned the relative rule and were making their choices based on relative length as opposed to surface area.

#### Generalization test of relative line length rule

**Fig. 3:**
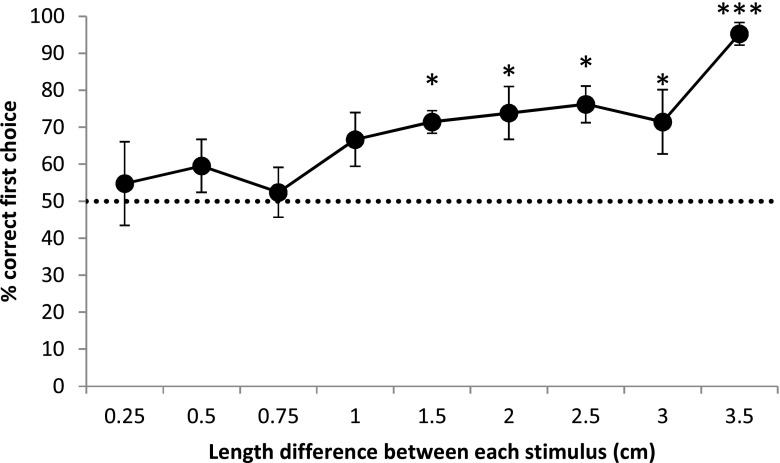
Mean ± *SE* (%) correct first choices for the discrimination threshold test. The dotted line represents chance level (.5). One-sample Poisson rate test: ^*^p < .05, ^***^p < .001

Figure 3 reveals successful generalization to all types of generalization stimuli (Pair 1, 83.3% ± 9.6, *p* = .003; Pair 2, 92.9% ± 4.9, *p* < .001; Pair 3, 88.1% ± 9.4, *p* = .001), except those in which the contingencies were reversed (Pair 4, 52.4% ± 10.5, *p* = .442). This suggests that, while the animals had learned a relative rule, they had also learned information about the contingencies associated with specific stimuli.

#### Discrimination threshold test

**Fig. 4:**
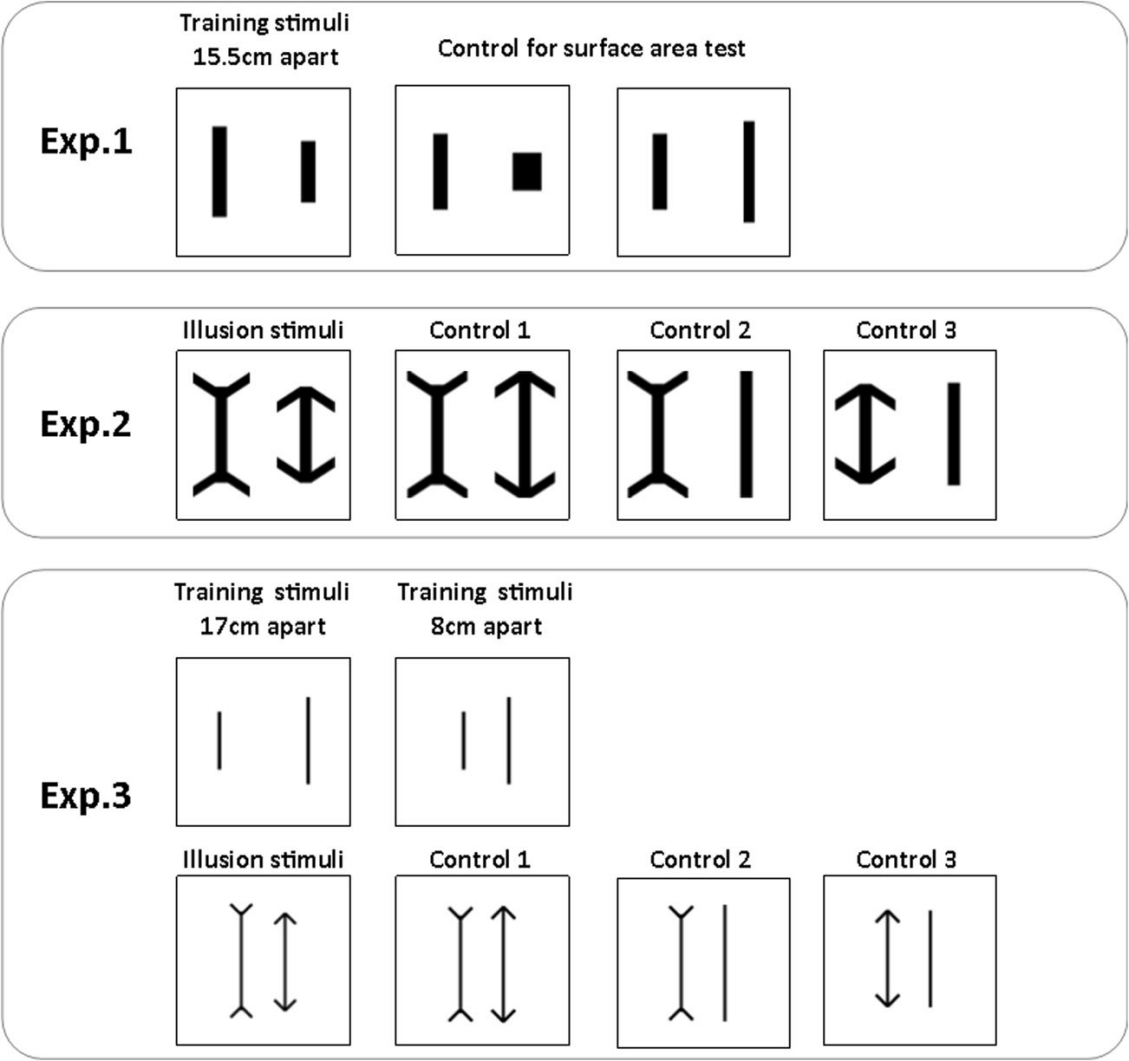
Training, test, and control stimuli used for Experiments 1, 2, and 3. In Experiment 1, the discrimination training stimuli were presented 15.5 cm apart. The control test for surface area paired the original line lengths with stimuli that were longer or shorter but equal in surface area. The Experiment 2 test stimuli were also presented 15.5 cm apart. The Experiment 2 and 3 Müller-Lyer tests each included three control arrangements that were used for each line length tested. Control 1 was one of the original inward-pointing arrows paired with an outward-pointing arrow equal in overall length; Control 2 was an original inward-pointing arrow paired with a line equal in overall length; Control 3 was an original outward-pointing arrow paired with a line equal in overall length. In Experiment 3, the discrimination training stimuli were presented 17 cm apart, which was then reduced to 8 cm apart. The Experiment 3 test stimuli were presented 8 cm apart

Dogs successfully discriminated between line lengths that differed by 1.5 cm; however, they were unable to differentiate anything less than this (Fig. 4).

#### Summary

These results revealed that the dogs were readily able to learn to discriminate between line lengths using a relational rule, something that has been observed in a wide variety of species (Manabe, Murata, Kawashima, & Asahina, 2009; Moll & Nieder, 2014). Dogs’ performance during the discrimination threshold test demonstrated that their visual capabilities, during such tasks, closely compare with those of penguins and sharks (Fuss et al., 2014; Manabe et al., 2009), and are in contrast to those of pigeons and humans, which far exceed dogs’ performance level (Schwabl & Delius, 1984).

## Experiment 2

Once the dogs had learned the line length discrimination in Experiment 1, modified versions of these lines were used to investigate their perception of the Müller-Lyer illusion. Although susceptibility to the Müller-Lyer illusion has been reported in other mammals (Suganuma et al., 2007; Tudusciuc & Nieder, 2010) in the same directions as in humans, dogs have previously shown no susceptibility or a reversed susceptibility to other visual illusions (Byosiere, Feng, Rutter, et al., 2017; Byosiere, Feng, Woodhead, et al., 2017; Miletto Petrazzini et al., 2017). Thus, we had no a-priori predictions for dogs’ responses to the illusory stimuli.

### Method

#### Subjects and apparatus

The subjects were the seven dogs from Experiment 1. The apparatus and experimental setup used were identical to those used during Experiment 1.

#### Procedure

The training procedure and stimuli were identical to those of Experiment 1.

#### Müller-Lyer test

To investigate whether the animals perceived the Müller-Lyer illusion, test trials were introduced in which the training stimuli that measured 10.5, 12.5, 14.5, and 16.5 cm tall and 2 cm wide had arrowheads added to the ends of each line. In each test trial, lines of the same length were presented, one of which had arrows pointing outward, and the other of which had arrows pointing inward; this was done so as to create the Müller-Lyer illusion (Fig. 1).

#### Control tests

To accurately assess the dogs’ perception of the illusion, we then presented them with three test conditions, which were applied to each of the four line lengths. The procedure for all tests was identical to that used during the tests sessions in Experiment 1. Animals received six exposures to each test pairing over the course of 24 sessions, resulting in a total of 96 test trials, 32 for each condition. These were presented randomly and counterbalanced across sessions.

##### Comparison of Müller-Lyer figures with equal overall lengths

For this control test, we presented a line with inward-pointing arrowheads paired with a line with outward-pointing arrowheads. The lines were of different lengths, however, so that the global images (including the arrowheads) had the same overall length (Fig. 1; Control 1).

##### Müller-Lyer figures paired with lines of equal length

These stimuli were designed as follows.

- *Inward-pointing arrowheads.* For this control test, we presented a line with inward-pointing arrowheads paired with a line of equal overall length (Fig. 1; Control 2).
- *Outward-pointing arrowheads.* For this control test, we presented a line with outward-pointing arrowheads paired with a line of equal overall length (Fig. 1; Control 3).

### Results

#### Müller-Lyer illusion test

**Fig. 5:**
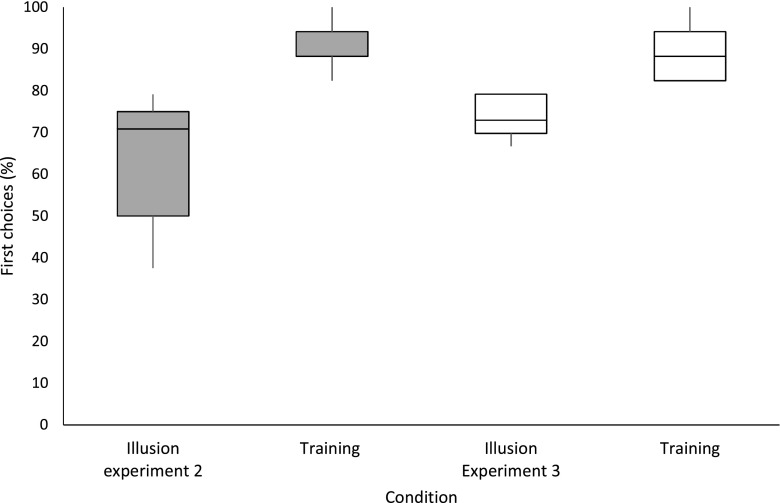
First choice responses for illusion probe trials and training trials during the probe testing for Experiments 2 and 3. For the training trials, first choices depict performance according to a dog’s trained contingencies (longer/shorter); for the illusion stimuli, first choices depict performance in relation to selecting the stimulus with inward-pointing arrowheads as being longer and the stimulus with outward-pointing arrowheads as being shorter, for a direct comparison between human and dogs’ responses to the illusion

Dogs chose the line with inward-pointing arrowheads as being longer and the line with outward-pointing arrowheads as being shorter 64.3% ± 2.9% of the time (*p* = .007; Fig. 5), suggesting that they may perceive the illusion in the same way that humans do.

#### Control tests

##### Comparison of Müller-Lyer figures of equal overall length

**Fig. 6:**
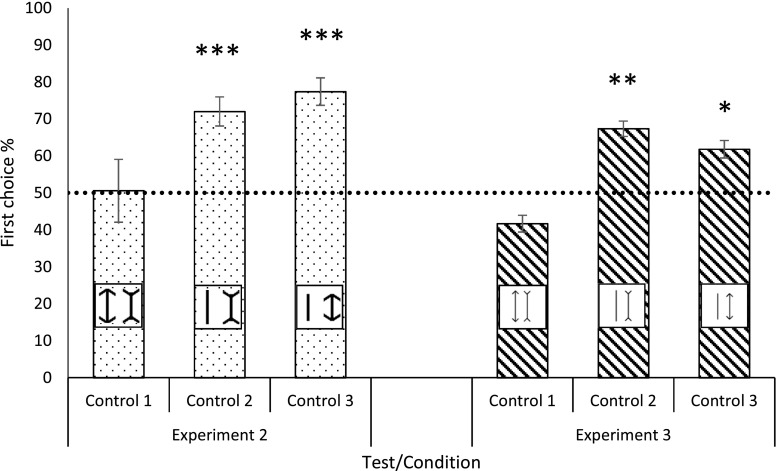
Mean first choices ± *SE*s (%) during Control Tests 1, 2, and 3 of Experiment 2 (dotted bars) and Experiment 3 (diagonal-lined bars). Bars depict dogs’ choices of the left stimulus within each pair. Control 1: A line with outward-pointing arrowheads paired with a stimulus of equal length composed of a line with inward-pointing arrowheads. Control 2: A line length paired with an equal-length stimulus consisting of a line with inward-pointing arrowheads. Control 3: A line length paired with an equal-length stimulus composed of a line with outward-pointing arrowheads. The dotted line represents chance (.5). One-sample Poisson rate test: ^*^p < .05, ^**^p < .01, ^***^p < .001

The animals discriminated between physical line length differences (arrowheads excluded), according to the trained contingencies, with an accuracy of 45.8% ± 5.1%; this did not differ from chance (*p* = .792). When comparing their choices of stimuli, we found no difference in the numbers of times they chose the outward-pointing arrowheads, 50.6% ± 8.5%, as compared with the inward-pointing arrowheads, 49.4% ± 8.5% (*p* = .471; Fig. 6).

##### Müller-Lyer figure paired with an equal line length

The results for the different stimuli follow.

- *Inward-pointing arrowheads*. Dogs discriminated between physical line length differences (arrowheads excluded), according to the trained contingencies, with an accuracy of 60.1% ± 10.1% (*p* = .039). However, when examining their specific choices of stimuli, we found they chose the line stimulus significantly more, 72% ± 3.9%, than the Müller-Lyer figure (*p* < .001; Fig. 6).
- *Outward-pointing arrowheads*. These results revealed that the dogs chose the line stimulus over the Müller-Lyer figure 77.4% ± 3.7% of the time (*p* < .001; Fig. 6).

#### Summary

Our data from the Müller-Lyer illusion tests suggest that dogs perceive the illusion in a manner similar to how humans do; however, the control tests revealed that dogs appeared to use the features of the stimulus, rather than the illusion itself, when making their choices.

## Experiment 3

There is evidence to suggest that line thickness, arrowhead angle, and the distance between comparison lines can affect the magnitude of the Müller-Lyer illusion (Pepperberg et al., 2008; Pressey & Dilollo, 1978). Thus, to investigate whether our findings were the result of specific aspects of our stimuli, in Experiment 3 the thickness of the shafts was reduced, stimuli were moved closer together, and the arrowheads were changed to measure 45°, in an attempt to increase the magnitude of the illusion.

### Method

#### Discrimination training

The dogs were trained to discriminate stimuli on the basis of relative length. The stimuli consisted of 12 bars measuring 3–14 cm tall (1-cm increments) and 0.5 cm wide. These were arranged in pairs that differed in length by at least 4 cm. Each dog was assigned 12 stimulus pairs. The stimuli were initially presented 17 cm apart, as in Experiment 1, until a criterion of > 80% correct first choices across three consecutive sessions was achieved. This distance was then reduced to 8 cm. From this point onward, all stimuli, including both test and control stimuli, were presented 8 cm apart. The contingencies for each dog remained unchanged. Otherwise, training was identical to that in Experiment 1.

#### Tests

All tests were identical to those used in Experiment 2, except that the novel stimuli and positions were used (see Fig. 1).

#### Data analysis

The data were processed in the same way as in Experiment 2.

### Results

#### Discrimination training

Six dogs reached the learning criterion in 15.5 ± 3 training sessions. There was no difference in performance between the dogs that were reinforced for the longer (*N* = 3) or for the shorter (*N* = 3) stimulus (Mann–Whitney *U* = 4.5, *p* = 1). One dog (Ivo) failed to reach the learning criterion within 60 sessions, and thus did not participate any further.

When the lines were moved closer together, all six dogs reached the learning criterion in 7 ± 1.9 training sessions.

#### Müller-Lyer illusion test

Dogs chose the line with inward-pointing arrowheads as being longer and the line with outward-pointing arrowheads as being shorter 73.6% ± 2.5% of the time (*p* < .001; Fig. 5), suggesting that they may perceive the illusion in a manner similar to how humans do. There was no significant increase in performance on the Müller-Lyer illusion test between Experiment 2 and Experiment 3, Wilcoxon: *Z* = – 1.612, *p* = .107.

#### Control tests

##### Comparison of Müller-Lyer figures of equal overall length

The dogs discriminated between physical line length differences (arrowheads excluded), according to their trained contingencies, with an accuracy of 45.8% ± 5.1% (*p* = .697). When comparing their choices of stimuli, we found that they chose the inward-pointing arrowheads more often, 58.3% ± 2.3%, than the outward-pointing arrowheads, 41.7± 2.3, but this difference was nonsignificant (*p* = .090; Fig. 6).

##### Müller-Lyer figures paired with lines of equal length

The results for the different stimuli follow.

- *Inward-pointing arrowheads.* Dogs discriminated between physical line length differences (arrowheads excluded), according to their trained contingencies, with an accuracy of 71.5% ± 7.9% (*p* < .001). When comparing their choices of stimuli, we found they chose the line stimulus significantly more, 67.4% ± 2.1%, than the Müller-Lyer figure (*p* < .003; Fig. 6).
- *Outward-pointing arrowheads.* Dogs chose the line stimulus over the Müller-Lyer figure (61.8% ± 7.7% vs. 38.2% ± 2.4%, *p* = .029; Fig. 6).

#### Summary

The results of the experiment did not differ from those of Experiment 2, suggesting that the dogs’ failure to pass the control test was not the result of specific aspects of our stimuli, but rather, appears to be due to information processing.

## General discussion

The findings of this work reveal that dogs are readily able to learn to discriminate line lengths on the basis of a relative rule. Furthermore, our data appear to suggest that dogs perceive the Müller-Lyer illusion in the same way that humans do; that is, they chose the line with inward-pointing arrowheads as being longer and the line with outward-pointing arrowheads as being shorter. Similar results have been reported in other mammals (Suganuma et al., 2007; Tudusciuc & Nieder, 2010), birds (Nakamura et al., 2006; Pepperberg et al., 2008), and fish (Sovrano et al., 2015). However, not all of these studies explicitly controlled for the possibility that the animals were responding to the global stimulus as opposed to perceived line length. Our control tests revealed that dogs were likely using global stimulus features (global size) rather than line length to make the discrimination.

This finding is in contrast to work investigating the Ebbinghaus–Titchener illusion, in which dogs perceived both the illusory-contour and classic versions of this illusion (Byosiere, Feng, Woodhead, et al., 2017). During the classic version, dogs responded to the illusion in the reverse direction, as compared to humans. If dogs were using global stimulus size to make this discrimination, as opposed to perceiving the illusion, then the data obtained would be exactly the same; however, during this study overall stimulus size was specifically controlled for, making this interpretation highly unlikely. Since dogs have shown no susceptibility to the Delboeuf illusion (Byosiere, Feng, Woodhead, et al., 2017; Miletto Petrazzini et al., 2017) and do not appear to be deceived by the Müller-Lyer illusion, either, it may be that they are sensitive to illusory contours but are less sensitive to size illusions, similarly to goldfish and sharks (Fuss et al., 2014; Wyzisk & Neumeyer, 2007). In that case, dogs may be less sensitive to the size difference induced by the Müller-Lyer illusion, which is considered more subtle (Byosiere, Feng, Woodhead, et al., 2017; Fuss et al., 2014) than the Ebbinghaus–Titchener illusion (Byosiere, Feng, Woodhead, et al., 2017).

This may be related to dogs’ overall ability to discriminate between very small size differences. Dogs’ visual acuity is relatively poor, compared with other species (Miller & Murphy, 1995) and may be too low to detect the very slight line length deception evoked by the Müller-Lyer illusion (Fuss et al., 2014). In our discrimination threshold test, dogs were unable to reliably discriminate between line lengths that differed from one another by < 1.5 cm. This is comparable with a number of other species (penguins: Manabe et al., 2009; sharks: Fuss et al., 2014). In contrast, pigeons have successfully discriminated between length differences of 0.2 mm, which is similar to that observed in humans (Schwabl & Delius, 1984). Both pigeons (Nakamura et al., 2006; Nakamura et al., 2009) and humans (e.g., Hesse, Franz, & Schenk, 2016; Pressey & Dilollo, 1978) have been found to be susceptible to the Müller-Lyer illusion whereas the aforementioned sharks were not. Together with our results, this suggests the possibility that animals with relatively poor visual acuity may not be capable of perceiving the Müller-Lyer illusion due to its comparatively weak effect (Byosiere, Feng, Woodhead, et al., 2017; Fuss et al., 2014). This fits with other recent work, which has suggested that some visual tasks given to dogs may exceed the capacity of their visual perception (Pongrácz, Ujvári, Faragó, Miklósi, & Péter, 2017).

During Experiment 1, we demonstrated that dogs can be successfully trained to discriminate lines according to the abstract rule of relative length. Previously, they have been shown to discriminate relative quantities (using food: Baker et al., 2012; Miletto Petrazzini & Wynne, 2016, 2017; Ward & Smuts, 2007; using opponent group size: Bonanni et al., 2011). Dogs successfully generalized their learning to novel stimuli and stimulus combinations. However, when they were faced with a stimulus that had always been an S+ during training, paired with a novel stimulus that reversed this contingency (according to the relative rule), neither cue was salient enough to control the dogs’ behavior. Reinforcement history has been shown to greatly affect dogs’ responses even in the presence of explicit task information, where the accumulation of previous learning overrides other relevant information (Ashton & De Lillo, 2011).

Overall, our findings reveal that dogs can apparently perceive the Müller-Lyer illusion. However, when appropriate controls were used, they revealed that dogs used the global stimulus information rather than judging perceived line length. These findings suggest that visual acuity may play a crucial role in perceiving the illusion. This research has implications for our understanding of dog perception and is likely to be an important consideration in the appropriate training of working animals.

### Author note

We thank the owners of the animals that participated in this study, Ludwig Huber for providing access to equipment, and Tatjana Hoehfurtner, Ferenc Igali, and Michael Steurer for their technical support.
